# 945. Rewriting the Record: Transforming Data Management for a Smarter System

**DOI:** 10.1093/jbcr/irag033.512

**Published:** 2026-04-06

**Authors:** Carey K Lamphier, Jasmin Mercedes, Pamela Vanderberg, Laura S Johnson, Lauren B Nosanov

**Affiliations:** Grady Memorial Hospital, Atlanta, GA; Grady Memorial Hospital, Atlanta, GA; Grady Memorial Hospital, Atlanta, GA; Grady Memorial Hospital / Emory School of Medicine / Morehouse School of Medicine, Atlanta, GA; Emory University School of Medicine, Atlanta, GA

## Abstract

**Introduction:**

Data-driven decision making is essential to delivering high quality patient care. The ability to efficiently capture, manage, and analyze data is critical. Our large, urban, American Burn Association (ABA) verified burn center cares for 3500 outpatients and over 600 inpatients annually. Recent expansions made by the ABA to the Burn Care Quality Platform (BCQP) requirements for patient data and encounter capture required our Burn Process Improvement (PI) team to mature our existing strategies.

**Methods:**

We sought to implement a system to simplify documentation and support identification of trends and actionable items in a more streamlined and robust approach. We reviewed all data points currently captured across all workflows then evaluated existing resources within our health system to determine capabilities and limitations with a plan for a period of iterative refinements. A model that had achieved demonstrated success was chosen.

Initially, data were dispersed across nearly 20 unrelated spreadsheets, which we consolidated into three centralized HIPAA compliant databases already enmeshed in institutional cloud data sharing systems. System setup facilitated direct integration of data from prehospital notifications, BCQP registry, and the centralized Burn PI tracking mechanism. Existing protocols and practices were reviewed and adjusted to account for workflow adjustments. Most queries are now directly transmitted and can be responded to within the platform, obviating the need for external communication and documentation.

**Results:**

Enhanced efficiency was immediately evident in reduced email-based queries and collaboration. Reduced redundancy enables easier report generation and increased ability to focus time on interpretation of findings, creation of action plans, project development, and research activities by PI team members. With improved extraction accuracy, we paired PI team efforts with review of clinical documentation practices, including addressing areas noted for documentation inaccuracy or extraction difficulty. Engagement in regional and national academic fora through formalized research has afforded higher scrutiny of accumulated data. Significant improvement in accuracy has been appreciated. The impact of these changes is monitored through regular meetings.

**Conclusions:**

Long-term success of data capture, management, and analysis is rooted in constant attention to opportunities for improvement and new and emerging technological solutions. Strong commitment to teamwork and mentorship within the PI team along with onboarding processes designed to address data management technology ensures long-term sustainability.

**Applicability of Research to Practice:**

Improved data and ease of communication with our clinical and management partners allows us to address unanticipated challenges. More effective and efficient workflow leads to better data, increased ability to make decisions, and more holistic patient care.

**Funding for the study:**

N/A.

